# Arthroscopic Hip Capsular Repair Improves Patient-Reported Outcome Measures and Is Associated With a Decreased Risk of Revision Surgery and Conversion to Total Hip Arthroplasty

**DOI:** 10.1016/j.asmr.2023.100800

**Published:** 2023-10-06

**Authors:** Soshi Uchida, Kazuha Kizaki, Maharaj S. Arjuna, Yoichi Murata, Yoshiharu Shimozono, Kazutomo Miura, Koichi Nakagawa, Atsuo Nakamae, Toshiyasu Nakamura, Tadahiro Sakai, Kazuhiko Kikugawa, Tatsuo Mae, Eiichi Tsuda, Harukazu Tohyama

**Affiliations:** a Department of Orthopaedic Surgery, Wakamatsu Hospital of the University of Occupational and Environmental Health, Kitakyushu, Japan; b Department of Surgery, Division of Arthroscopy & Sports Medicine, Dalhousie University, Halifax, Nova Scotia, Canada; c Temerty Faculty of Medicine, University of Toronto, Toronto, Ontario, Canada; d Department of Orthopaedic Surgery, Kyoto Shimogamo Hospital, Kyoto, Japan; e Department of Orthopaedic Surgery, Kensei Hospital, Hirosaki, Japan; f Department of Orthopaedic Surgery, Toho University Sakura Medical Center, Sakura, Chiba, Japan; g Department of Orthopaedic Surgery, Graduate School of Biomedical and Health Sciences, Hiroshima University, Hiroshima, Japan; h Department of Orthopaedic Surgery, School of Medicine, International University of Health and Welfare, Tokyo, Japan; i Department of Orthopaedic Surgery, Toyota Memorial Hospital, Nagoya, Japan; j Department of Orthopaedic Surgery, Mazda Hospital, Hiroshima, Japan; k Department of Orthopaedic Surgery, Osaka University Graduate School of Medicine, Osaka, Japan; l Department of Rehabilitation Medicine, Hirosaki University Graduate School of Medicine, Hirosaki, Japan; m Faculty of Health Sciences, Hokkaido University, Sapporo, Japan

## Abstract

**Purpose:**

To perform a systematic review to assess the effect of capsular repair compared with nonrepair on patient-reported outcome measures (PROMs) and conversion to total hip arthroplasty (THA) after hip arthroscopy in patients with femoroacetabular impingement syndrome.

**Methods:**

We initially searched the Cochrane Central Register of Controlled Trials (CENTRAL), MEDLINE, EMBASE, and PubMed databases, as well as ongoing clinical trials (https://clinicaltrials.gov), on December 15, 2022. The eligibility criteria were randomized controlled trials (Level Ⅰ) and prospective comparative studies (Level II) of patients who underwent capsular repair and nonrepair via hip arthroscopy with a minimum follow-up period of 2 years. We registered this protocol a priori on PROSPERO (identification No. CRD42021239306). We assessed the risk of bias using the Methodological Index for Non-randomized Studies (MINORS) appraisal tool.

**Results:**

This review included 5 studies with a total of 639 patients (270 with capsular repair [average age, 35.4 years; 41% female patients] and 369 with nonrepair [average age, 37.3 years; 38% female patients]). In the included studies, surgical procedures consisting of labral repair and pincer or cam osteoplasty were performed via hip arthroscopy. The modified Harris Hip Score was measured in all the included studies, and the standardized mean difference in PROMs for capsular repair versus nonrepair in the included studies was 0.42 (95% confidence interval [CI], 0.20 to 0.63). A sensitivity analysis of randomized controlled trials achieved consistent results (standardized mean difference in PROMs, 0.31; 95% CI, 0.02 to 0.60). Capsular repair was not associated with a reduction in revision surgery (risk difference, –0.02; 95% CI, –0.06 to 0.03; 26 of 270 patients with capsular repair vs 42 of 369 with nonrepair) but was associated with a reduction in conversion to THA (risk difference, –0.05; 95% CI –0.09 to –0.01; 12 of 270 patients with capsular repair vs 38 of 369 with nonrepair). The average Methodological Index for Non-randomized Studies (MINORS) score in the included studies was 20.

**Conclusions:**

Patients who undergo capsular repair in conjunction with other arthroscopic hip preservation techniques have better PROMs and a lower incidence of THA conversion.

**Level of Evidence:**

Level II, systematic review of Level I and II investigations.

Hip arthroscopy is a minimally invasive surgical procedure performed to treat intra-articular hip disorders, such as femoroacetabular impingement syndrome (FAIS), and extra-articular pathologies.^1^ The vast majority of hip arthroscopists prefer capsulotomy or capsular release to improve visualization and instrument accessibility; however, it is unclear how large the capsular repair should be to be beneficial during hip arthroscopy. The hip capsular ligaments reinforce the stability of the hip^2^; however, release of these ligaments can facilitate arthroscopic management to treat intra-articular pathologies. A biomechanical study has shown that capsular repair reinforces hip instability.^3^ Previous systematic reviews have shown the benefit of capsular closure in hip arthroscopy^3^, ^4^, ^5^, ^6^, ^7^, ^8^, ^9^, ^10^, ^11^, ^12^, ^13^, ^14^ (Appendix Table 1). However, it is inconclusive how much capsular closure with hip arthroscopy has an impact on patient-reported outcome measures (PROMs) among high-level studies (Levels I and II). The complete PICO (Population, Intervention, Comparison, and Outcome)–format research question is shown in Appendix 1.

This study aimed to perform a systematic review to assess the effect of capsular repair compared with nonrepair on PROMs and conversion to total hip arthroplasty (THA) after hip arthroscopy in patients with FAIS. We hypothesized that capsular repair would significantly improve PROMs and decrease postoperative complications (e.g., incidence of revision surgery and conversion to THA) compared with capsular nonrepair in hip arthroscopy.

## Methods

### Comprehensive Literature Search

We registered this protocol a priori on PROSPERO (identification No. CRD42021239306) in April 2021. In April 2021 and again in December 2022, we conducted a comprehensive literature search for all relevant articles using 4 electronic databases: Cochrane Central Register of Controlled Trials (CENTRAL) (Cochrane Library, 2022), MEDLINE (1946 to December 15, 2022), EMBASE (1974 to December 15, 2022) and PubMed (1990 to December 15, 2022). The PubMed search was limited to the period after 1989 because hip arthroscopy has only been widely adopted since the 1990s.^15^ We also examined ongoing trials using a database of clinical trials (https://clinicaltrials.gov).

Each search strategy is shown in Appendix 2. In the manual search, we screened the reference lists of previous meta-analyses and relevant studies for additional articles not identified through the electronic search. We chose the following key search terms: (Hip∗) AND (arthroscopy OR arthroscopic OR arthroscop∗) AND (capsule OR capsular OR capsul∗).

We adopted a 3-step screening process (title screening, abstract screening, and full-text screening) to select eligible articles. After the duplicate articles were removed, 2 board-certified orthopaedic surgeons (K. Kizaki and Y.M.) independently performed the title and abstract screenings. If either reviewer included an article during title or abstract screening, the article was moved to the next stage for screening. During the full-text screening, discrepancies were resolved through discussion and consensus with the senior author (S.U.). For each study, 2 reviewers (K. Kizaki and Y.M.) independently extracted the following data: article type, journal, authors, publication year, country, study design, number of patients, number of female patients, age, follow-up period, PROMs, surgery time, and complications.

### Criteria for Study Inclusion

In this review, we included studies that compared capsular repair and nonrepair in hip arthroscopy. Capsular repair was considered present when complete capsular repair was performed using any surgical procedure. Studies were not excluded based on patient age or disease resulting in a surgical indication. The eligibility criteria were as follows: Level I and II studies comparing capsular repair versus nonrepair, patients treated with hip arthroscopic surgery for conditions including FAIS, and studies presenting at least 1 primary outcome (i.e., PROM) (Appendix Table 2). The exclusion criteria were cadaveric studies, patients with hip dysplasia, noncomparative studies, studies with no description of capsular procedures, studies including the same cohort as another study (studies including more patients were prioritized), and studies with less than 2 years of follow-up data.

The primary outcome was PROMs (e.g., modified Harris Hip Score [mHHS] and Hip Outcome Score). The secondary outcome was complications such as surgical-site infection, symptomatic deep vein thrombosis, or pulmonary embolism; revision surgery; and conversion to THA.

### Risk-of-Bias Assessment

Two epidemiologists with expertise in systematic reviews (K. Kizaki and M.A.) independently evaluated the risk of bias using the Methodological Index for Non-randomized Studies (MINORS) appraisal tool.^16^

### Synthesis of Results

Dichotomous outcomes (i.e., complications) were expressed as risk differences with 95% confidence intervals (CIs). Continuous outcomes (i.e., PROMs) were expressed as mean differences between the capsular repair and nonrepair groups. We preferred to calculate effect size measures (standardized mean difference [SMD]) when studies used different instruments to assess the primary outcome (i.e., PROMs). When multiple measurement scales were used in a study, we adopted the most commonly used measurement scale in the included studies in a quantitative data synthesis (meta-analysis).

We analyzed outcomes according to the modified intention-to-treat method without imputation. When data were not expressed as mean and standard deviation but were expressed as median, minimum-maximum range, or interquartile range, we estimated the mean and standard deviation in accordance with Wan et al.^17^

We pooled the included studies using the generic inverse variance method for continuous outcomes and the Mantel-Haenszel method for dichotomous outcomes in Review Manager (RevMan 5, The Nordic Cochrane Centre, Copenhagen, Denmark). Either a fixed-effect or random-effect model was used depending on heterogeneity. The heterogeneity among pooled studies was assessed using the χ^2^ test, with the level of statistical significance set at *P* < .10, and the *I*^2^ statistic. The *I*^2^ value was assessed as follows: 0% to 40%, not important; 30% to 60%, moderate; 50% to 90%, substantial; and 75% to 100%, considerable.^18^ In addition, we considered the variance in the point estimate and overlap in the CIs. We prespecified and conducted sensitivity analyses based on the PROMs of randomized controlled trials (RCTs). We also prespecified and performed subgroup analysis according to the 2 most common capsular repair techniques. For the primary outcome measures (PROMs), we evaluated the degree of the effect size in reference to Cohen et al.,^5^ with an SMD of 0.2 to 0.5 defined as small; 0.5 to 0.8, medium; and greater than 0.8, large.

## Results

### Results of Search

We screened a total of 2,048 articles (MEDLINE, 582; EMBASE, 902; PubMed, 517; and CENTRAL, 47). After the removal of duplicates, we screened the titles of 1,458 articles and the abstracts of 487 articles. We identified 5 articles from the reference lists of previous meta-analyses and implemented a full-text screening process. As shown in Appendix Table 2, we also performed critical appraisal of the previous systematic reviews answering the following clinical question: Does capsular repair yield preferable outcomes compared with capsule nonrepair in hip arthroscopy? From the database of clinical trials, we found 4 studies in progress; however, the results were not presented. The ongoing trials are shown in Appendix Table 3.

**Fig 1:**
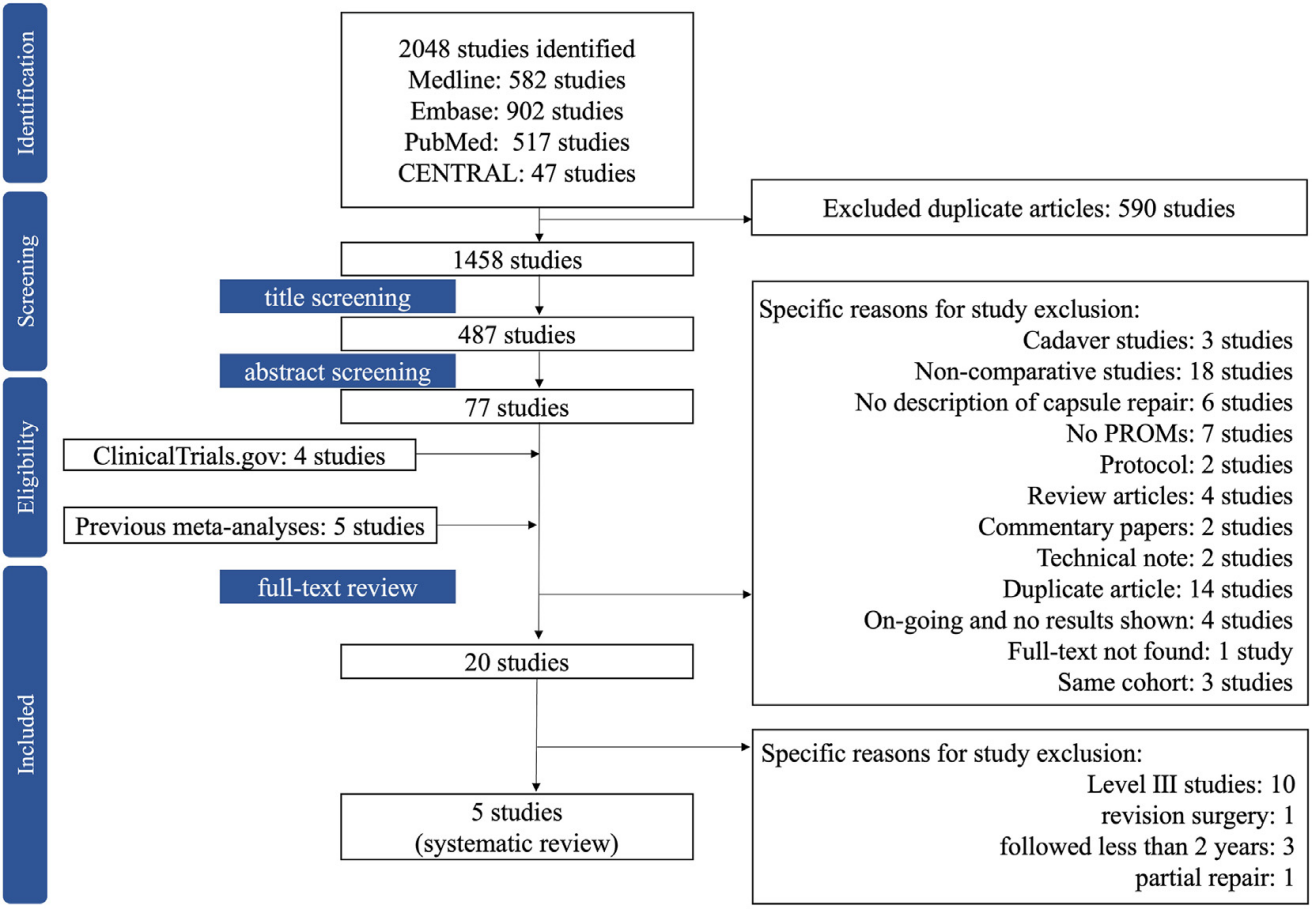
PRISMA (Preferred Reporting Items for Systematic Reviews and Meta-analyses) flowchart. (CENTRAL, Cochrane Central Register of Controlled Trials; PROMs, patient-reported outcome measures.)

Overall, 5 studies were included in this systematic review.^19^, ^20^, ^21^, ^22^, ^23^ Details regarding the screening process are illustrated in the PRISMA (Preferred Reporting Items for Systematic Reviews and Meta-analyses) flowchart^24^ in Figure 1.

### Included and Excluded Studies

**Appendix Fig 1:**
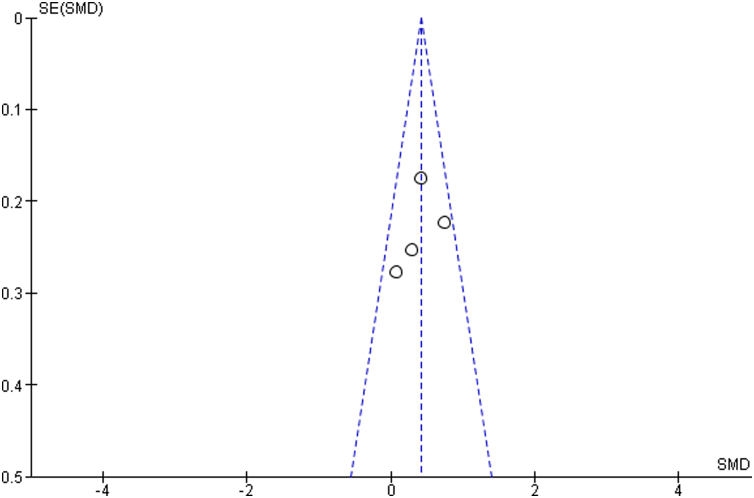
Funnel plot of standardized mean difference (SMD) in patient-reported outcome measures in included studies. (SE, standard error.)

We ultimately included 5 studies with a total of 639 patients (270 in capsular repair group and 369 in nonrepair group) (Appendix Fig 1). All studies were published between 2017 and 2022.^25^, ^26^, ^27^, ^28^ Details of the included studies are provided in Table 1. The most common surgical indication in the included studies was FAIS resistant to conservative treatment. In the included studies, the surgical indications were no advanced arthritis (Tönnis grade 0 or 1) with younger age (average, 36.5 years). Postoperative clinical outcomes in each study are presented in Appendix Table 2.

**Table 1:** Characteristics of Included Studies

Authors	Article Type	Journal	Year	Country	Study Design	Level of Evidence	Patients, n		Female Patients, n		Age, yr (mean)		Follow-up (mean)	
							CR	CNR	CR	CNR	CR	CNR	CR	CNR
Economopoulos et al.^21^	Full paper	AJSM	2019	United States	RCT	Ⅰ	50	100	19	42	35.2	37.8	2 yr	2 yr
Atzmon et al.^20^	Full paper	JHPS	2019	Israel	Prospective	Ⅱ	35	29	14	13	38.1	37.6	40.4 mo	60.7 mo
Sugarman et al.^22^	Full paper	OJSM	2021	United States	RCT	Ⅰ	26	26	22	8	31.8	33.7	2 yr	2 yr
Ashberg et al.^19^	Conference (AANA)	—	2017	United States	Prospective	Ⅱ	115	172	NS	NS	NS	NS	5 yr	5 yr
Tahoun et al.^23^	Full paper	KSSTA	2022	Spain	Prospective	Ⅱ	44	42	9	11	35.6	38.2	5 yr	5 yr

AANA, Arthroscopic Association of North America; AJSM, *American Journal of Sports Medicine*; CNR, capsular nonrepair; CR, capsular repair; JHPS, *Journal of Hip Preservation Surgery*; NS, not shown; OJSM, *Orthopedic Journal of Sports Medicine*; KSSTA, *Knee Surgery, Sports Traumatology, Arthroscopy*; RCT, randomized controlled trial.

### Risk of Bias in Included Studies

In the included studies, the mean MINORS score was 20. MINORS scores are presented in Appendix Table 4. All of the included studies followed up the patients for 2 years or more.

### Patient-Reported Outcome Measures

**Fig 2:**
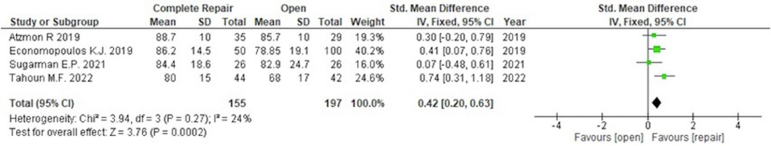
Standardized (Std) mean differences in patient-reported outcome measures for capsular repair versus nonrepair in hip arthroscopy among Level I and II studies. (CI, confidence interval; IV, inverse variance; SD, standard deviation.)

**Fig 3:**
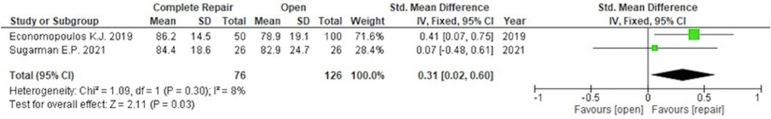
Standardized (Std) mean differences in patient-reported outcome measures for capsular repair versus nonrepair in hip arthroscopy among randomized controlled trials. (CI, confidence interval; IV, inverse variance; SD, standard deviation.)

**Fig 4:**
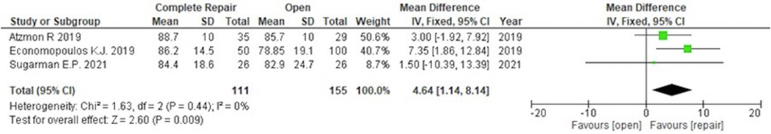
Mean differences in modified Harris Hip Score for capsular repair versus nonrepair in hip arthroscopy among Level I and II studies. (CI, confidence interval; IV, inverse variance; SD, standard deviation.)

The most common PROM was the mHHS (5 studies), followed by the Hip Outcome Score–Activities of Daily Living (HOS-ADL) scale (4 studies). On qualitative synthesis, the study of Ashberg et al.^19^ showed the superiority of capsular repair according to PROMs (Appendix Table 2). In the meta-analysis of studies reporting at least 1 PROM, the SMD (0.42; 95% CI, 0.20 to 0.63) indicated that capsular repair yielded favorable PROMs compared with nonrepair in hip arthroscopy, as shown in Figure 2. When we limited the analysis to RCTs, 2 studies were included,^21^^,^^22^ and the SMD was again significant (0.31; 95% CI, 0.02 to 0.60)^5^ (Fig 3). The mean difference in the mHHS showed the superiority of capsular repair over nonrepair in hip arthroscopy (mean difference, 4.64; 95% CI, 1.14 to 8.14) (Fig 4).

### Subgroup Analysis of PROMs Between Interportal and T-Shaped Capsulotomy

We prepared a subgroup analysis based on the surgical techniques of capsulotomy a priori (i.e., T-shaped capsulotomy vs interportal capsulotomy); however, there was no pooled study on T-shaped capsulotomy. Therefore, we did not perform subgroup analysis of PROMs between interportal and T-shaped capsulotomy.

### Complications

**Fig 5:**
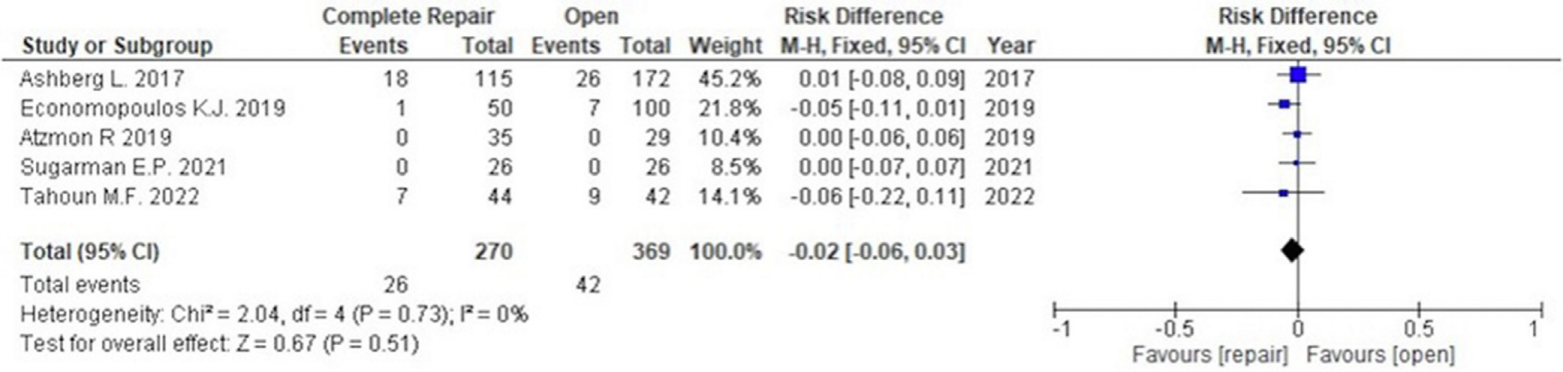
Incidence of revision surgery for capsular repair versus nonrepair in hip arthroscopy. (CI, confidence interval; M-H, Mantel-Haenszel test.)

**Fig 6:**
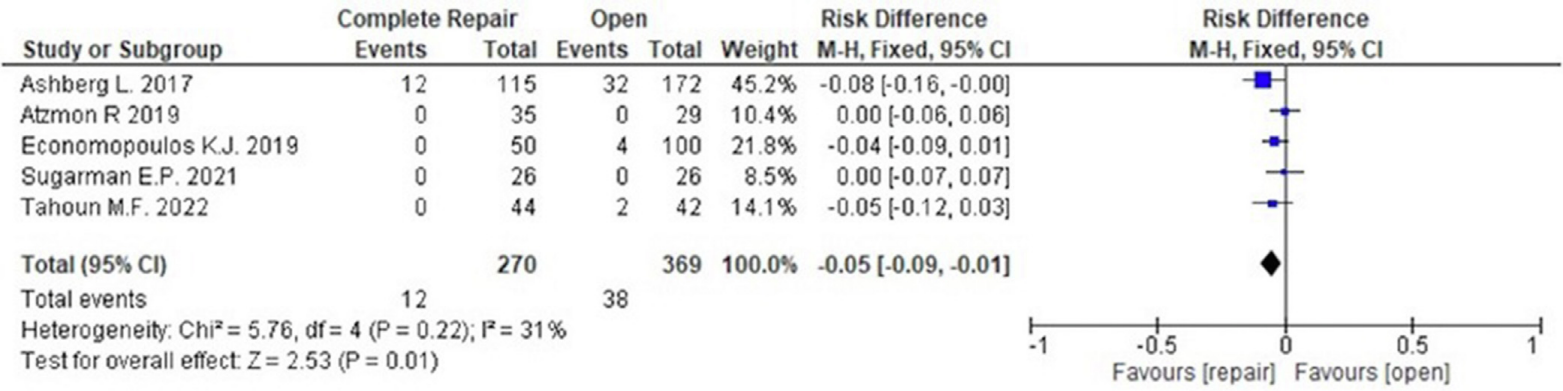
Incidence of conversion to total hip arthroplasty for capsular repair versus nonrepair in hip arthroscopy. (CI, confidence interval; M-H, Mantel-Haenszel test.)

The most frequently monitored complication was the number of revision surgical procedures and conversions to THA. Regarding revision surgery, 5 studies with 639 patients were included. The incidence of revision surgery was not associated with capsular repair in hip arthroscopy (risk difference, –0.02; 95% CI, –0.06 to 0.03; 26 of 270 patients with capsular repair vs 42 of 369 with nonrepair), as shown in Figure 5. Regarding conversion to THA, 5 studies with 639 patients were included with surgical indication (nonarthritis; Tönnis grade 0 or 1). The incidence of conversion to THA was significantly associated with capsular repair (pooled mean follow-up, 47.3 months) when compared with nonrepair (pooled mean follow-up, 47.8 months) (risk difference –0.05; 95% CI, –0.09 to –0.01; 12 of 270 patients with capsular repair vs 38 of 369 with nonrepair), as shown in Figure 6. In the pooled populations, no severe complications such as symptomatic deep venous thrombosis, pulmonary embolism, or deep surgical-site infection were reported.^29^

## Discussion

The most important finding of this study was that capsular repair in hip arthroscopy was statistically significantly superior to nonrepair in terms of PROMs, a finding that was maintained when the analysis was limited to RCTs; however, the effect size was small. The incidence of revision surgery was not higher with nonrepair than with capsular repair during hip arthroscopy, but the incidence of conversion to THA was lower with capsular repair. We identified 6 previous meta-analyses in the literature before running our search.^4^^,^^7^^,^^8^^,^^12^, ^13^, ^14^ At the time point when we updated the search on December 15, 2022, an additional 5 meta-analyses (quantitative synthesis) and 1 systematic review (qualitative synthesis) had already been published.^3^^,^^5^^,^^9^, ^10^, ^11^ In our comprehensive search, we covered all the comparative studies included in the previous meta-analyses.

Riff et al.^13^ found that interportal capsulotomy and T-shaped capsulotomy are the most common techniques for capsular repair. Various types of suture techniques for capsular repair have recently been introduced.^30^^,^^31^ To identify the superiority of interportal versus T-shaped capsulotomy, we prepared a subgroup analysis; however, because there was no pooled study on T-shaped capsulotomy, this analysis was not completed. Recently, arthroscopic capsular reconstruction has been developed.^32^ Further studies are required to evaluate clinical differences between interportal capsulotomy, T-shaped capsulotomy, and capsular reconstruction.^33^ Capsular repair is rational in the stabilization of hip joints. Our meta-analysis showed the positive impact on PROMs and the association with a reduction in the incidence of conversion to THA; these results support the practical application of capsular repair in hip arthroscopy. Hip arthroscopy is associated with small risks of symptomatic deep venous thrombosis (0.09%) and deep surgical-site infection (0.02%)^34^; therefore, severe complications were not present in the pooled populations.

The most noteworthy strength of our review is its robust methodology: This review’s protocol was registered on PROSPERO prior to searching and data collection. This review followed the guidelines of the *Cochrane Handbook for Systematic Reviews of Interventions*. Two board-certified orthopaedic surgeons specializing in hip surgery and arthroscopy conducted the searches, and 2 epidemiologists with educational backgrounds in health research methodologies performed the data synthesis (meta-analysis) and evidence assessment according to the GRADE (Grading of Recommendations Assessment, Development and Evaluation) approach. Another strength is the generalizability to the target population treated with hip arthroscopy. In the pooled analysis, the most common surgical indication was FAIS resistant to conservative treatment, which is the main surgical indication for hip arthroscopy.

### Limitations

Several limitations are present in this review. First, our review only included 2 RCTs. RCTs are the gold-standard study design because this design prevents selection bias. Second, we encountered limitations in accessing the gray literature. To ensure a broad search, we completed searches in 4 databases and 1 online clinical trials platform, but a residual risk of reporting bias may be present. Third, the surgical procedure of capsular repair has potential risk of performance bias: therefore, capsular repair may be a surrogate procedure with skillful procedures. Fourth, capsular nonrepair is applicable in several situations. In cases with hip joint stiffness, capsular release may be intentionally selected to avoid postoperative stiffness. Therefore, whether capsular repair versus nonrepair should be performed in hip arthroscopy may not be a simple issue; rather, it depends on each case. Finally, this systematic review has revised and modified the original search methodology, which may have influenced our results.

## Conclusions

Patients who undergo capsular repair in conjunction with other arthroscopic hip preservation techniques have better PROMs and a lower incidence of THA conversion.

## Figures and Tables

**Figure:**
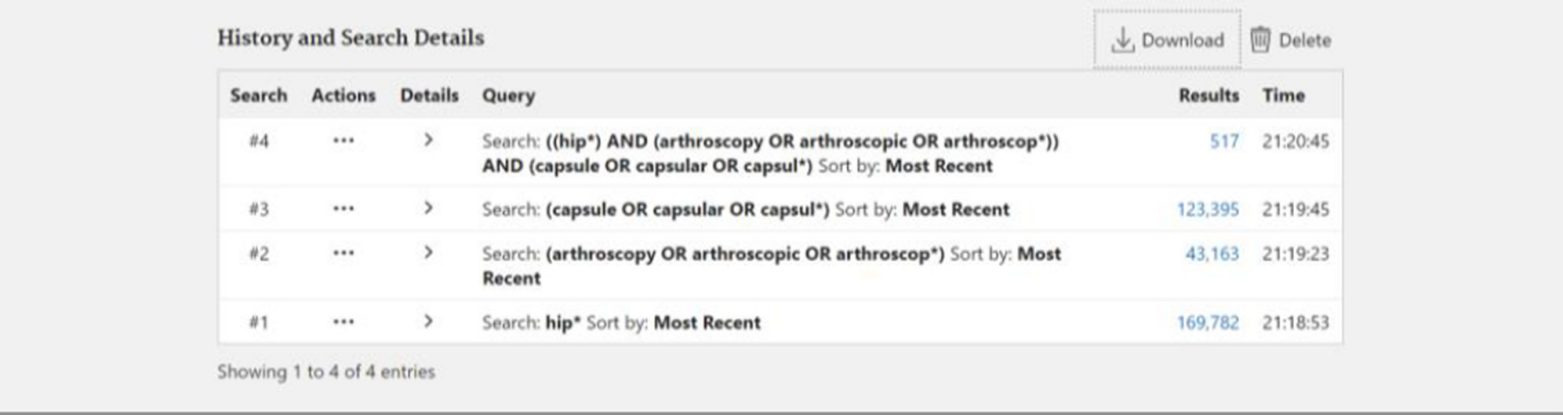


**Appendix Table 1:** Critical Appraisal of Previous Systematic Reviews

Authors	Journal	Year	Advantages	Limitations
Riff et al.^13^	*Arthroscopy*	2019		Noncomparative studies
Ortiz-Declet et al.^12^	*Arthroscopy*	2018		Preliminary review, updated by Jimenez et al.^3^ (2021)
Westermann et al.^14^	Iowa Orthop J	2018		Search performed in December 2016
Acuna et al.^4^	J Orthop	2020		Insufficient search, pooling only 659 patients in terms of mHHS
Lin et al.^8^	Hip Int	2020		Insufficient search, pooling only 294 patients in terms of revision rate
Liu et al.^7^	Orthop Surg	2020		Incorrect interpretations of forest plots, leading to opposite conclusion
Morris et al.^10^	COR	2021		Only performed qualitative synthesis
Jimenez et al.^3^	*Arthroscopy*	2021		Biomechanical studies; primary outcomes should be important to patients
Owens et al.^11^	*Arthroscopy*	2021		Insufficient searching of standardized mean difference with mHHS; duplicate study cohorts
Kunze et al.^6^	OJSM	2021	Restricted to prospective comparative studies	Shown registration identification No. was not applied in PROSPERO; surrogate marker as primary outcome
Cohen et al.^5^	KSSTA	2022	Robust methodology; comprehensive search (pooled 4,744 patients)	Noncomparative studies
Looney et al.^9^	AJSM	2022	Registered study protocol a priori	Many duplicated study cohorts; noncomparative studies

AJSM, *American Journal of Sports Medicine*; COR, *Current Orthopedic Practice*; Hip Int, *HIP International*; Iowa Orthop J, *Iowa Orthopaedic Journal*; J Orthop, *Journal of Orthopedics*; KSSTA, *Knee Surgery, Sports Traumatology, Arthroscopy*; mHHS, modified Harris Hip Score; OJSM, *Orthopedic Journal of Sports Medicine*; Orthop Surg, *Orthopedic Surgery*.

**Appendix Table 2:** PROMs and Surgical Procedures in Included Studies

Authors	Year	Disease	PROM														Arthroscopic Portal					Capsulotomy	Tönnis Grade	
			mHHS	HOS-ADL	HOS-SS	NAHS	WOMAC	VAS for Pain	Satisfaction	iHOT-12	VR-12	SF-12 or SF-36	Vail hio score	UCLA	NRS	EQ-5D	Anterior	Anterolateral	Mid-anterior	Mid-lateral	Distal AL Accessory		CR	CNR
Economopoulos et al.^21^	2019	FAIS	✓	✓	✓												✓	✓	✓			Interportal	0 or 1	0 or 1
Atzmon et al.^20^	2019	FAIS	✓	✓	✓				✓									✓	✓			Interportal	0	0
Sugarman et al.^22^	2021	FAIS	✓	✓	✓			✓		✓	✓							✓	✓			Interportal	NS∗	NS∗
Ashberg et al.^19^.	2017	FAIS	✓				✓					✓		✓								NS	0 or 1	0 or 1
Tahoun et al.^23^	2022	FAIS	✓	✓	✓		✓	✓		✓												Interportal and T-shaped	0 or 1	0 or 1

EQ-5D, the EuroQol 5 dimension 5 level; HOS-ADL, Hip Outcome Score- activities of daily living; HOS-SS, Hip Outcome Score- Sport Scale; iHOT-12, International Hip Outcome Tool Outcome Tool-12; NAHS, Nonarthritis Hip Score; NRS, Numerical Rating Scale; NS, not shown; PROM, patient-reported outcome measure; SF-12, 12-item short form survey; SF-36, 36-item short form survey; UCLA, University of Calfornia Los Angels; VAS, visual analog scale; VR-12, Veterans RAND 12; WOMAC, Western Ontario and McMaster Universities Osteoarthritis Index.

∗ Sugarman et al. excluded cases with a narrow femoroacetabular joint space (<2 mm) on plain radiographs.

**Appendix Table 3:** Ongoing Clinical Trials

Study Identification No.∗	Principle Investigator	Country	Status	Estimated Study Completion Data	Status on July 15, 2022
NCT03372564	Aaron Krych	United States	Recruiting	December 2023	Not yet reported
NCT03158454	Martin Lind	Denmark	Recruiting	December 2021	Not yet reported
NCT03751618	Nicolas Bonin	France	Recruiting	July 2021	Not yet reported
NCT02990234	Omer Mei-Dan	United States	Completed	October 2017	Partially reported (results published [*J Hip Preserv Surg* 2018;5:349-356] but PROMs not reported)

∗ In reference to clinicaltrials.gov (https://clinicaltrials.gov/).

**Appendix Table 4:** MINORS Scores for Included Studies

	Atzmon et al.^20^	Economopoulos et al.^21^	Sugarman et al.^22^	Ashberg et al.^19^	Tahoun et al.^23^
	2019	2019	2021	2017	2022

Clearly stated aim	2	2	2	2	2
Inclusion of consecutive patients	2	2	2	0	0
Prospective collection of data	2	2	2	2	2
Endpoints appropriate to aim of study	2	2	2	2	2
Unbiased assessment of study endpoint	0	2	2	0	2
Follow-up period appropriate to aim of study	2	2	2	2	2
Loss to follow-up < 5%	2	2	1	1	1
Prospective calculation of study size	0	2	2	0	0
Adequate control group	2	2	2	2	2
Contemporary groups	2	2	2	1	2
Baseline equivalence of groups	2	2	2	2	2
Adequate statistical analysis	2	2	2	2	2
Total	20	24	23	16	19

MINORS, Methodological Index for Non-randomized Studies.

**Table:** Debatable article should be included in the update search on July 15, 2022

Article	Reason for Exclusion
Li Z-y, Hu G-f, Jin Z-g, et al. Capsular healing in interportal and periportal capsulotomy methods of hip arthroscopy. *Orthop Surg* 2021;13:1863-1869.	Size of capsulotomy was significantly different between capsular repair and nonrepair groups because capsular nonrepair was performed for cases with periportal capsulotomy.

## Appendix 1: PICO (Population, Intervention, Comparison, and Outcome)–Format Research Question

Does capsule repair improve PROMs and decrease postoperative complications (e.g., the risks of revision surgery and conversion to THA) compared with nonrepair in hip arthroscopy?

## Appendix 2: Search Strategy

### MEDLINE Search

Ovid MEDLINE: Epub Ahead of Print, In-Process & Other Non-Indexed Citations, Ovid MEDLINE Daily and Ovid MEDLINE <1946-Present>

1 "hip∗,".mp. [mp=title, abstract, original title, name of substance word, subject heading word, floating subheading word, keyword heading word, organism supplementary concept word, protocol supplementary concept word, rare disease supplementary concept word, unique identifier, synonyms] 421883

2 (arthroscopy or arthroscopic or arthroscop∗).mp. [mp=title, abstract, original title, name of substance word, subject heading word, floating subheading word, keyword heading word, organism supplementary concept word, protocol supplementary concept word, rare disease supplementary concept word, unique identifier, synonyms] 38537

3 (capsule or capsular or capsul∗).mp. [mp=title, abstract, original title, name of substance word, subject heading word, floating subheading word, keyword heading word, organism supplementary concept word, protocol supplementary concept word, rare disease supplementary concept word, unique identifier, synonyms] 123370

4 1 and 2 and 3 540

5 limit 4 to yr="1990 -Current" 536

### EMBASE Search

Embase Classic+Embase <1947 to 2021 December 15>

1 "hip∗,".mp. [mp=title, abstract, heading word, drug trade name, original title, device manufacturer, drug manufacturer, device trade name, keyword, floating subheading word, candidate term word] 597042

2 (arthroscopy or arthroscopic or arthroscop∗).mp. [mp=title, abstract, heading word, drug trade name, original title, device manufacturer, drug manufacturer, device trade name, keyword, floating subheading word, candidate term word] 51773

3 (capsule or capsular or capsul∗).mp. [mp=title, abstract, heading word, drug trade name, original title, device manufacturer, drug manufacturer, device trade name, keyword, floating subheading word, candidate term word] 176479

4 1 and 2 and 3 831

5 limit 4 to yr="1990 -Current" 828

### CENTRAL Search

Comment:

ID Search Hits

#1 (hip∗) AND (arthroscopy OR arthroscopic OR arthroscop∗) AND (capsule OR capsular OR capsul∗) (Word variations have been searched) 47

## Supplementary Data

ICMJE author disclosure forms
